# Redox Mechanisms of AVS022, an Oriental Polyherbal Formula, and Its Component Herbs in Protection against Induction of Matrix Metalloproteinase-1 in UVA-Irradiated Keratinocyte HaCaT Cells

**DOI:** 10.1155/2013/739473

**Published:** 2013-09-19

**Authors:** Thanyawan Pluemsamran, Pinpat Tripatara, Rattana Phadungrakwittaya, Pravit Akarasereenont, Tawee Laohapand, Uraiwan Panich

**Affiliations:** ^1^Department of Pharmacology, Faculty of Medicine Siriraj Hospital, Mahidol University, Bangkok 10700, Thailand; ^2^Center of Applied Thai Traditional Medicine, Faculty of Medicine Siriraj Hospital, Mahidol University, Bangkok 10700, Thailand

## Abstract

Ayurved Siriraj HaRak (AVS022) formula has been used for topical remedy of dermatologic disorders. Oxidative stress induced by ultraviolet (UV) A irradiation could be implicated in photoaged skin through triggering matrix metalloproteinase-1 (MMP-1). We, therefore, explored the antioxidant mechanisms by which AVS022 formulation and its individual components protected against UVA-dependent MMP-1 upregulation in keratinocyte HaCaT cells. TLC analysis revealed the presence of multiple phenolics including gallic acid (GA) in the AVS022 extracts. We demonstrated that pretreatment with the whole formula and individual herbal components except *T. triandra* protected against increased MMP-1 activity in irradiated HaCaT cells. Moreover, all herbal extracts and GA, used as the reference compound, were able to reverse cytotoxicity, oxidant production, glutathione (GSH) loss, and inactivation of catalase and glutathione peroxidase (GPx). *F. racemosa* was observed to yield the strongest abilities to abolish UVA-mediated induction of MMP-1 and impairment of antioxidant defenses including GSH and catalase. Our observations suggest that upregulation of endogenous antioxidants could be the mechanisms by which AVS022 and its herbal components suppressed UVA-stimulated MMP-1 in HaCaT cells. In addition, pharmacological actions of AVS022 formula may be attributed to the antioxidant potential of its components, in particular *F. racemosa*, and several phenolics including GA.

## 1. Introduction

Demands for alternative medicines including herbal remedies continue to increase. Herbal treatment for dermatologic diseases and cosmetic problems has existed for thousands of years [1]. Ayurved Siriraj HaRak (AVS022) formula, a Thai polyherbal formula consisting of 5 medicinal plants, has been used in Thai traditional medicine for the remedy of skin disorders. Thus, exploring pharmacological activities of the AVS022 polyherbal formula and its constituent herbs is of significance in order to gain scientific evidence on the efficacy and safety of traditional herbal medicine. The AVS022 formula is composed of the root extracts of 5 herbs, *Capparis micracantha* DC., *Clerodendrum indicum* L., *Harrisonia perforata* Merr., *Ficus racemosa* L., and *Tiliacora triandra* (Colebr.) Diels. Previous *in vitro* and *in vivo* studies of *F. racemosa*, a medicinal plant used in Indian ayurvedic medicine, reported that it exerted several pharmacological actions including anti-inflammatory, anticancer, and antioxidant activities [2–4]. Additionally, various phytochemical constituents including racemic acid and alkaloids isolated from *F. racemosa* and *T. triandra*, respectively, which are also the herbal components of AVS022 formula, were demonstrated to possess biological activities [5, 6]. 

Ultraviolet A (UVA) (315–400 nm) has been recognized as a primary environmental cause of photodamage and premalignant changes of the skin cells through cytotoxicity of keratinocytes and activation of metalloproteinase-1 (MMP-1), a major collagenolytic enzyme generated by keratinocytes and fibroblasts. Since MMP-1 is contributed to skin cell damage and collagen fragmentation affecting the skin's structural integrity [7], development of dermatological products that effectively suppress MMP-1 at cellular and molecular levels could be a targeting strategy for photoaging prevention. Medicinal plants and phytochemicals including phenolic acids providing antioxidant properties have been observed to abrogate damaging effects of UVA on the skin through inhibition of activity and expression of MMP-1 in keratinocytes or fibroblasts [8–10]. We previously reported that impaired capacity of antioxidant defenses including catalase, glutathione peroxidase (GPx), and glutathione (GSH) involved UVA-stimulated MMP-1 activity, and therefore, upregulation of endogenous antioxidants may represent mechanisms underlying photoprotective effects of phytochemicals [10]. We, thus, assessed antioxidant mechanisms of AVS022 extracts, its 5 plant components, and gallic acid (GA), an antioxidant phenolic present in the formula, in protecting against UVA-dependent cell toxicity and MMP-1 augmentation by assessing cellular oxidant generation, GSH level, and catalase and GPx activities in immortalized human keratinocyte (HaCaT) cells.

## 2. Materials and Methods

### 2.1. Materials

Human keratinocyte cell line (HaCaT) from Cell Lines Service (CLS, Heidelberg, Germany) was a kind gift from Professor Pa-thai Yenchitsomanus, Department of Research and Development, Faculty of Medicine Siriraj Hospital, Mahidol University. Dulbecco's modified Eagle's media (DMEM) were purchased from Invitrogen (NY, USA), and chemicals and reagents of the highest quality available were obtained from Sigma-Aldrich (MO, USA or Germany). 

### 2.2. Preparation of AVS022 Formula Extracts

AVS022 composed of 20% (w/w) of each herb; *H. perforate*, *C. micracantha*, *C. indicum*, *F. racemosa*, and *T. triandra*, was prepared by Unit of Thai Herbal Pharmaceuticals of Center of Applied Thai Traditional Medicine, Faculty of Medicine Siriraj Hospital, Mahidol University, Thailand. All plant materials were purchased from Tai-hua-jan drugstore and authenticated by two experienced Thai traditional practitioners using macroscopic identification and organoleptic techniques which based on anatomical characteristics of the individual plant parts and color, fracture, smell, or taste. Then the specimens were sorted, washed, oven-dried, and crushed. The powdered drug was extracted by dynamic maceration method. One hundred grams of the powdered herb was weighed and placed in a container with 1 L of 80% (v/v) ethanol. The mixture was constantly stirred with magnetic stirrer for 10 minutes, and then the liquid was filtered and the marcs were pressed. The clarified liquid was evaporated under reduced pressure by rotary evaporator and kept frozen overnight prior to lyophilization. The extraction procedure for individual plant was the same with previously referred to procedure. One hundred milligrams of the lyophilized powder was accurately weighed and dissolved in 1 mL of 80% ethanol, mixed, and centrifuged at 15,000 rpm for 10 minutes at 4°C. The sample solution was filtered through a 0.2 **μ**m membrane filter and was used for thin layer chromatography (TLC) analysis.

### 2.3. TLC Fingerprinting of AVS022 Formula Extracts

**Figure 1:**
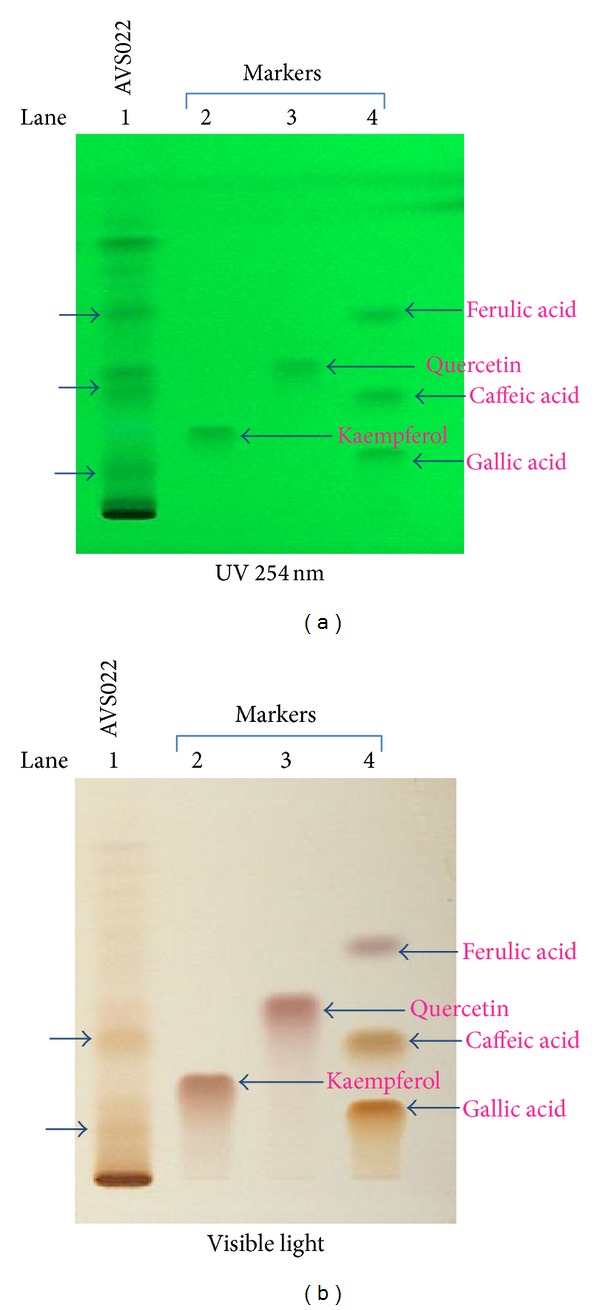
TLC fingerprinting of AVS022 extracts visualized under UV light (254 nm) (a) and visible light (after spraying with FBS) (b).

The filtrate of sample solution was loaded to TLC silica gel 60 F 254 (Merck, Germany) using sample applicator (Camag Linomat 5, Switzerland). Solvent system of hexane: ethyl acetate: acetic acid (31 : 14 : 5 v/v/v) was used as mobile phase for phenolic separation. The detection was examined under 254 nm UV light and visible light after spraying with fast blue salt (FBS). The identification of phenolics in AVS022 was carried out by comparing its TLC chromatogram with those of phenolic reference markers. Five phenolic reference markers including caffeic acid, ferulic acid, gallic acid, kaempferol, and quercetin were used. The TLC chromatograms showed the presence of caffeic acid, ferulic acid, and GA in the AVS022 extracts, and caffeic acid and GA were detected under both 254 nm UV light and visible light (after spraying with FBS) as shown in Figure 1. 

### 2.4. Cell Cultures and Treatment

HaCaT cells were cultured in DMEM/F12 medium supplemented with 10% fetal bovine serum (FBS) and 1% penicillin (100 units/mL)/streptomycin (100 **μ**g/mL) at 37°C in a humidified air of 5% CO_2_ (*P*_CO_2__ = 40 Torr) (a Forma Scientific CO_2_ Water-Jacketed Incubator). Cells were treated with the AVS022 extract; each component extract or GA used as the reference compound dissolved in 80% ethanol, and the final concentration of ethanol in culture medium did not exceed 0.05% (v/v). To assess photoprotective and antioxidant effects, cells were treated with herbal extracts at concentrations up to 60 **μ**g/mL and GA up to 5 **μ**g/mL for 30 min before UVA (330–400 nm) exposure. UVA intensity verification and selection of a UVA dose (4 J/cm^2^) and time point after irradiation were previously described [10]. GA was used as the reference phenolic in this study because it was shown to possess stronger inhibitory activity than that of caffeic acid and ferulic acid against oxidant formation in HaCaT cells exposed to UVA (4 J/cm^2^) (data not shown). Assays for cell viability, oxidant formation, GSH content, and antioxidant enzyme activities were carried out at 1 h time point and for MMP-1 activity at 24 h time point after UVA exposure.

### 2.5. Cell Lysate Preparation

Cells were harvested by centrifugation and lysed with buffer containing 50 mM Tris-HCl, 10 mM ethylene diaminetetraacetic acid (EDTA), 1% (v/v) Triton X-100, phenylmethylsulfonyl fluoride (PMSF) (100 mg/mL), and pepstatin A (1 mg/mL) in DMSO and leupeptin (1 mg/mL) in H_2_O, pH 6.8. The cells were centrifuged at 10,000 rpm for 10 min and the supernatant was then collected. Protein concentration was determined using the Bio-Rad Protein Assay Kit (Bio-Rad, Germany).

### 2.6. Cell Viability Assay

3-(4,5-Dimethylthiazol-2-yl)2,2 diphenyltetrazolium bromide (MTT) assay, based on the reduction of the yellow tetrazolium salt to purple formazan, was carried out to identify metabolically viable cells using a spectrophotometer (SpectraMax M2 of Molecular Device, CA, USA). Cell viability was expressed as a percentage of the control (100%, untreated cells without UV exposure).

### 2.7. MMP-1 Activity Assay by Zymography

Conditioned supernatants were collected for detecting MMP-1 activity using a zymography following the protocol previously reported [10]. Briefly, gelatinase substrate or samples were electrophoresized on nonreducing 10% sodium dodecyl sulphate-polyacrylamide gel electrophoresis (SDS-PAGE) containing gelatin. Then, gels were washed twice with 2.5% Triton X-100 to eliminate SDS and allow MMP-1 renaturation. The gels were then incubated in the reaction buffer containing 50 mM Tris-HCl, pH 8.8, supplemented with 1% Triton X-100, 10 mM CaCl_2_, 1 **μ**M ZnCl_2_, and 0.02% Na_3_N for 24 h at 37° to generate MMP-1-induced degradation of gelatin. The gels were stained with 0.006% Coomassie Blue G-250, and gelatinolytic activity of MMP-1 in the gel was visualized as nonstained bands on the blue background. The gels were scanned using a CAMAG TLC scanner (Muttenz, Switzerland), and integrated density of each band was analyzed to determine MMP-1 activity using the ImageMaster software (Hoefer Pharmacia Biotech). Data was represented as arbitrary densitometric units of MMP-1 activity per 1,000,000 cells.

### 2.8. Assay for Cellular ROS Formation

Dichlorofluorescein (DCFH) assay, based on the oxidation of nonfluorescent DCFH by intracellular ROS to fluorescent 2,7-DCF, was performed to evaluate formation of ROS. After cells were subjected to UVA (4 J/cm^2^), cells were incubated in DMEM without phenol red and loaded with 5 **μ**M DCFHDA for 1 h at 37°C. DCF fluorescence intensity was monitored for 30 min at excitation and emission wavelengths of 485 and 530 nm using a spectrofluorometer. The data are represented as a percentage of ROS production (relative fluorescence units, RFU) of the nontreated control cells without UVA exposure (100%).

### 2.9. Cellular Glutathione Content Assay

GSH level was measured using the fluorescent probe *o*-phthalaldehyde- (OPA-) based fluorometric method, principally by the reaction of GSH with OPA at pH 8. The cell extracts were prepared using 6.5% (w/v) trichloroacetic acid (TCA), and the GSH content assay was carried out as described previously [11]. The GSH content was detected by fluorescence intensity of the GSH-OPA adduct at excitation/emission wavelengths of 350/420 nm. GSH level was calculated using a GSH standard curve and was represented as **μ**M/mg protein.

### 2.10. Catalase Activity Assay

Catalase activity was measured colorimetrically by following the kit protocol from Cayman chemical (Ann Arbor, MI, USA). In principle, the enzyme reacted with methanol in the presence of H_2_O_2_ to produce the formaldehyde, which was determined spectrophotometrically at 540 nm using 4-amino-3-hydrazino-5-mercapto-1,2,4-triazole (Purpald). The standard curve was obtained using a formaldehyde standard. One unit of CAT activity was calculated as the amount of enzyme producing 1.0 nmol of formaldehyde per min at 25°C and represented as nmol/min/mg protein.

### 2.11. Glutathione Peroxidase Activity Assay

GPx activity was assessed by following manufacturer's protocol (Trevigen, Gaithersburg, MD, USA). The activity was indirectly measured by a coupled reaction with glutathione reductase (GR) causing reduction of oxidized glutathione. The oxidation of NADPH to NADP^+^ is accompanied by decreased absorbance at 340 nm as previously described [10]. One unit of GPx activity was determined as the amount of enzyme converting 1 nmol of NADPH to NADP^+^ per min at 25°C and represented as units/mg of protein.

### 2.12. Statistical Analysis

Data are represented as means ± standard error of the mean (SEM) of separate experiments (*n* ≥ 3) conducted on different days. The significance of individual treatment groups compared with irradiated groups was calculated using one-way analysis of variance (ANOVA) followed by Dunnett's test or by independent *t*-test (Student's; 2 populations) using Prism (GraphPad Software Inc., San Diego, CA, USA). 

## 3. Results

### 3.1. Inhibition of Cytotoxicity and MMP-1 Activation in Irradiated HaCaT Cells

**Figure 2:**
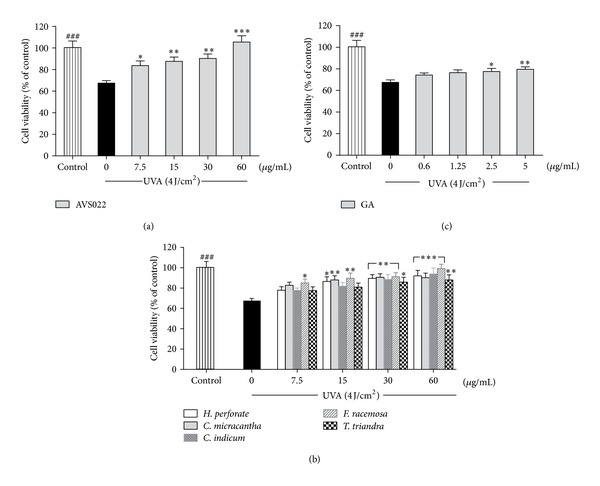
Inhibition of UVA- (4 J/cm^2^) induced HaCaT cell toxicity by the whole formula (a) and individual component herbs (b) of AVS022 and GA (c). Data was represented as mean ± SEM. The statistical significance of differences between the control and irradiated cells was assessed by Student's *t*-test and between UVA-irradiated and herb extracts- or GA-treated cells by one-way ANOVA followed by Dunnett's test. ^###^ *P* < 0.001 compared with irradiated cells. *P < 0.05; **P < 0.01; ***P < 0.001 compared with nontreated cells exposed to UVA.

Treatment of HaCaT with the AVS022 formula and each component herb at concentrations ranging from 7.5 to 60 **μ**g/mL and GA from 0.6 to 5 **μ**g/mL for 24 h without UVA exposure did not result in cytotoxicity (data not shown). Whereas a UVA dose of 4 J/cm^2^ caused a substantial decrease in cell viability by 32.95 ± 2.3% (*P* < 0.001) compared to nonirradiated cells, pretreatment with AVS022 (Figure 2(a)), its individual constituents (Figure 2(b)), and GA (Figure 2(c)) was able to significantly and dose-dependently hamper cytotoxicity induced by UVA irradiation. In addition, among 5 herbs, *F. racemosa* was observed to yield the greatest cytoprotective effect because it blocked UVA-mediated HaCaT toxicity at lower concentrations (7.5 **μ**g/mL) than those required for the 4 herbs.

**Figure 3:**
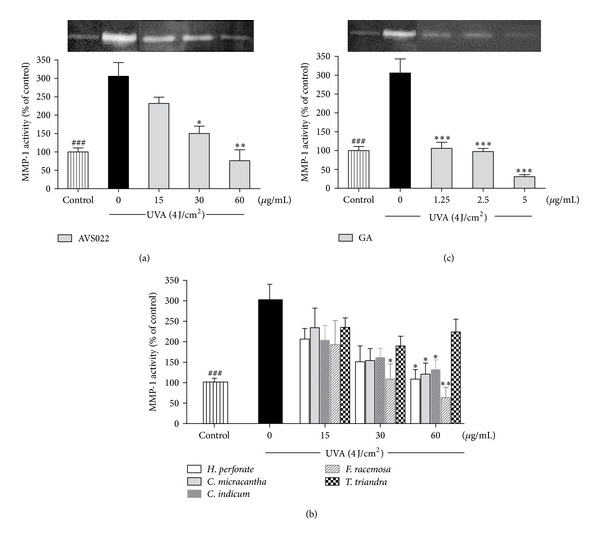
Inhibition of UVA-stimulated MMP-1 activity in HaCaT cells by the whole formula (a) and individual component herbs (b) of AVS022 and GA (c). Zymography analysis of secreted MMP-1 was performed as described in Section 2. Data was represented as mean ± SEM. The statistical significance of differences between the control and irradiated cells was evaluated by Student's *t*-test and between UVA-irradiated and herb extracts- or GA-treated cells by one-way ANOVA followed by Dunnett's test. ^###^ *P* < 0.001 compared with irradiated cells. *P < 0.05; **P < 0.01; ***P < 0.001 compared with nontreated cells exposed to UVA.

We further examined inhibition of UVA-stimulated MMP-1 activity by herb extracts and GA since MMP-1 is a major metalloproteinase for collagen destruction, a hallmark of photoaging. As shown in Figure 3, UVA (4 J/cm^2^) markedly stimulated activity of MMP-1 by 206 ± 2.3% (*P* < 0.001) compared to nonirradiated cells, although a significant and dose-dependent reduction of MMP-1 activity was observed in HaCaT cells pretreated with the whole formulation of AVS022 (Figure 3(a)), its component herbs but not *T. triandra *(Figure 3(b)), and GA (Figure 3(c)) compared with unpretreated cells following UV irradiation. In agreement with the photoprotective effect on HaCaT cell cytotoxicity, among 5 herb components of AVS022 formula*, F. racemosa* presented the strongest protective activity against UVA-induced enhanced MMP-1 activity since lower concentrations (30 **μ**g/mL) of *F. racemosa* than those of other 4 components were capable of suppressing MMP-1 stimulation.

### 3.2. Inhibition of ROS Formation and GSH Loss in Irradiated HaCaT Cells

**Figure 4:**
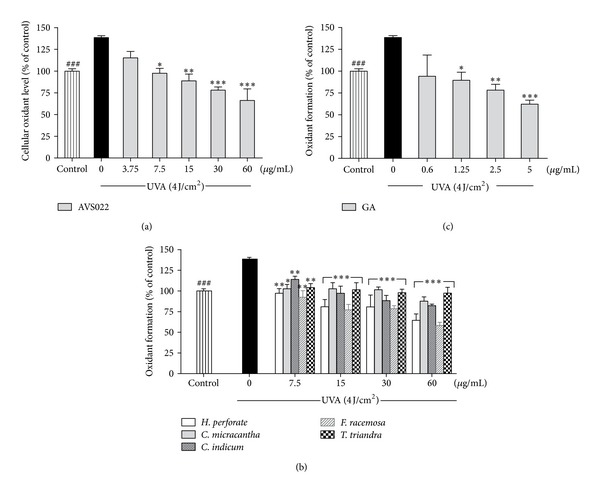
Inhibition of UVA-induced cellular ROS formation in HaCaT cells by the whole formula (a) and individual component herbs (b) of AVS022 and GA (c). The fluorescent DCF as an indicator of ROS formation was measured at 485 nm excitation and 530 nm emission as described in Section 2. Data were represented as a percentage of control (100%, nonirradiated and nontreated cells) using a microplate reader. The statistical significance of differences between the control and irradiated cells was determined by Student's *t*-test and between UVA-irradiated and herb extracts- or GA-treated cells by one-way ANOVA followed by Dunnett's test. ^###^ *P* < 0.001 compared with irradiated cells. *P < 0.05; **P < 0.01; ***P < 0.001 compared with nontreated cells exposed to UVA.

**Figure 5:**
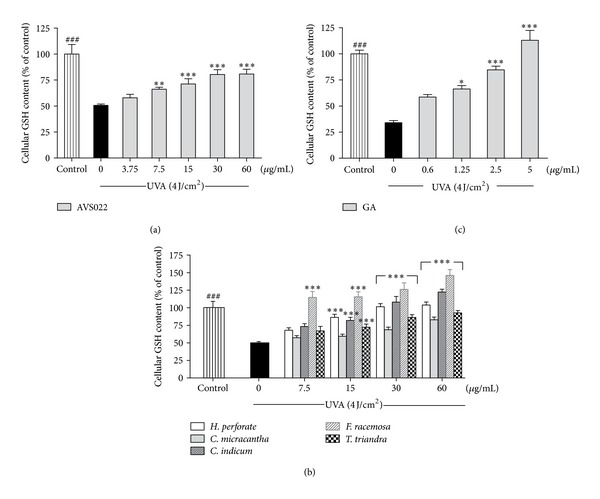
Inhibition of UVA-dependent GSH loss in HaCaT cells by the whole formula (a) and individual component herbs (b) of AVS022 and GA (c). GSH level was detected by fluorescence intensity of the GSH-OPA adduct at 350 nm excitation and 420 nm emission as described in Section 2. The statistical significance of differences between the control and irradiated cells was evaluated by Student's *t*-test and between UVA-irradiated and herb extracts- or GA-treated cells by one-way ANOVA followed by Dunnett's test. ^###^ *P* < 0.001 compared with irradiated cells. *P < 0.05; **P < 0.01; ***P < 0.001 compared with nontreated cells exposed to UVA.

Level of cellular ROS and GSH is an important marker to indicate cellular redox status. We assessed whether redox mechanisms were involved in the inhibitory effects of herb extracts studied and GA on UVA-dependent cytotoxicity and MMP-1 upregulation. Figures 4 and 5 demonstrated that, at 1 h postirradiation, UVA exposure (4 J/cm^2^) led to a substantial increase in ROS by 38.54 ± 2.1% (*P* < 0.001) and a dramatic decline in GSH level by 49.3 ± 1.3% (*P* < 0.001). In contrast, pretreatment of HaCaT cells with the whole formula (Figures 4(a) and 5(a)) and the individual components of AVS022 (Figures 4(b) and 5(b)) and GA (Figures 4(c) and 5(c)) caused a significant and dose-dependent inhibition of ROS formation and GSH loss as compared to unpretreated cells following UV irradiation. Furthermore, among all 5 components of AVS022, *F. racemosa* was shown to have the highest inhibitory effect on UVA-mediated reduced GSH content because the inhibitory concentrations (7.5 **μ**g/mL) of *F. racemosa* were lower than those required for the 4 herbs.

### 3.3. Inhibition of Catalase and Glutathione Peroxidase Inactivation in Irradiated HaCaT Cells

**Figure 6:**
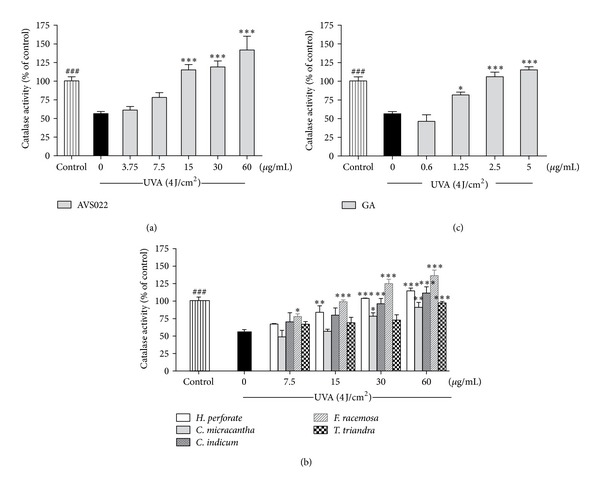
Inhibition of UVA-mediated catalase inactivation in HaCaT cells by the whole formula (a) and individual component herbs (b) of AVS022 and GA (c). The formaldehyde generated was determined spectrophotometrically with Purpald as a chromogen at 540 nm as described in Section 2. The statistical significance of differences between the control and irradiated cells was assessed by Student's *t*-test and between UVA-irradiated and herb extracts- or GA-treated cells by one-way ANOVA followed by Dunnett's test. ^###^ *P* < 0.001 compared with irradiated cells. *P < 0.05; **P < 0.01; ***P < 0.001 compared with nontreated cells exposed to UVA.

**Figure 7:**
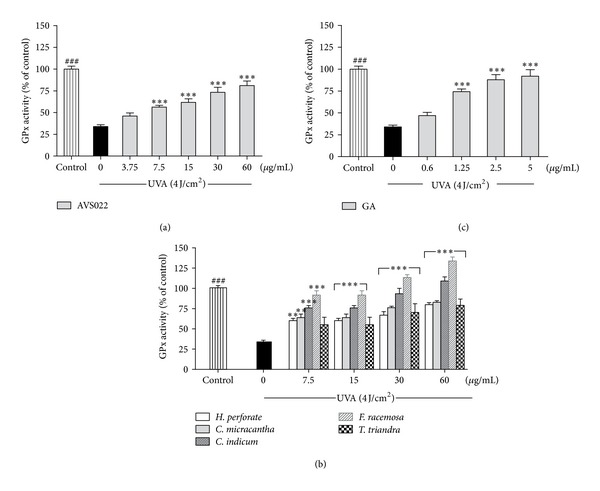
Inhibition of UVA-dependent GPx inactivation in HaCaT cells by the whole formula (a) and individual component herbs (b) of AVS022 and GA (c). GPx activity was evaluated as described in Section 2. The statistical significance of differences between the control and irradiated cells was determined by Student's *t*-test and between UVA-irradiated and herb extracts- or GA-treated cells by one-way ANOVA followed by Dunnett's test. ^###^ *P* < 0.001 compared with irradiated cells. *P < 0.05; **P < 0.01; ***P < 0.001 compared with nontreated cells exposed to UVA.

To further investigate redox mechanisms of herbal extracts studied and GA with respect to modulation of endogenous antioxidants, as shown in Figures 6 and 7, enzymatic assays revealed that, compared to nonirradiated control cells, UVA (4 J/cm^2^) irradiation drastically reduced catalase activity by 43.53 ± 7.7% (*P* < 0.001) and GPx activity by 66 ± 8.4% (*P* < 0.001). Nevertheless, addition of AVS022, each component herb, and GA prior to UVA exposure was able to dose-dependently reverse inactivation of both catalase and GPx compared to irradiated cells in the absence of herb extracts or GA. In agreement with our findings for cytotoxicity, MMP-1 activity, and GSH level, among all 5 components of AVS022, *F. racemosa* was shown to exert the most potent protection against UVA-dependent catalase inactivation.

## 4. Discussion

Development of herbs employed in a traditional medicine as promising photoprotective agents has gained considerable attention in dermatology research because pharmacologically active phytochemicals identified and isolated from several medicinal plants have been reported to yield antioxidant actions beneficial for the skin [12]. Since UVA irradiation-mediated oxidative stress of the skin is involved in keratinocyte toxicity and activation of MMP-1 accountable for photoaged skin, we, therefore, explored redox mechanisms of the whole formula and individual component herbs of AVS022 and GA, a reference phenolic compound, in protection against UVA-mediated cytotoxicity and MMP-1 induction in keratinocyte HaCaT cells. Our study demonstrated that AVS022, its constituent herbs, and GA significantly abrogated HaCaT cell toxicity mediated by UVA (4 J/cm^2^). Stimulation of MMP-1 activity by UVA was also suppressed by the whole formula and its individual herbal components except *T. triandra* component of AVS022 and GA. Previous studies reported that photooxidative stress is possibly involved in MMP-1 regulation in skin cells including keratinocytes [13, 14], and improving antioxidant defense system may thus be mechanisms underlying the photoprotective effects of phytochemicals ubiquitously present in medicinal plants. ROS accumulation in photoaged skins has been suggested to associate with increased MMP-1 expression, which could be reversed by promoting capacity of antioxidant defenses including catalase [15], GSH, and GPx [10, 16]. They are essential endogenous antioxidant defenses controlling redox balance accountable for protection against photooxidative stress in the keratinocytes and skin carcinogenesis [17, 18], and redox regulation of MMP-1 might, therefore, represent a strategy for photoaging prevention. We further investigated whether protective effects of the whole formula and each component of AVS022 and GA on UVA-mediated increased ROS formation and GSH depletion as well as inactivation of catalase and GPx were involved in the inhibition of MMP-1 activity. Our data indicated that pretreatment of irradiated HaCaT cells with the herbal extracts or GA abolished UVA-dependent GSH depletion and catalase and GPx inactivation. 

Since AVS022 is a polyherbal formulation composed of 5 medicinal plants, combinations of multiple active ingredients in different plants can make pharmacological action of AVS022 complex. We, thus, examined the modulation of MMP-1 and antioxidant defense capacity by AVS022 and individual component in our study. Zymographic analysis of MMP-1 activity showed that combination of 5 herbs did not yield synergistic protection against UVA-dependent enhanced MMP-1 activity and the *F. racemosa* component was primarily contributed to biological activities of the AVS022 formula because *F. racemosa *appeared to yield the most potent protective effect on UVA-induced MMP-1 activity. Furthermore, *F. racemosa* exerted the greatest abilities than the other 4 herbs to inhibit cytotoxicity, GSH depletion, and catalase inactivation mediated by UVA irradiation. In accordance with our study on free radical scavenging activity of all 5 herbal components using DPPH (1,1-diphenyl-2-picrylhydrazyl) assay, *F. racemosa *extracts possessed the strongest DPPH radical scavenging activity (data not shown). Our findings also suggested that protection against induction of MMP-1 by UVA appeared to correlate to the abilities of herbal extracts to improve the redox balance as *T. triandra*, which failed to suppress UVA-induced MMP-1 activation, had lower antioxidant activities than *F. racemosa* in restoring antioxidant defense system studied. Moreover, as reported in our previous study showing the protective effects of caffeic and ferulic acids on UVA-dependent MMP-1 stimulation in HaCaT cells, phenolic acids including caffeic, ferulic, and gallic acids identified in the AVS022 extracts could be possible active ingredients responsible for the biological activity of AVS022. Our data in this study is also consistent with previous studies for GA in modulation of redox system in different melanoma cell lines [19]. Nevertheless, further investigations concerning qualitative and quantitative analyses of phytochemicals present in the AVS022 formula and its component herbs using analytical techniques with high sensitivity and resolution (e.g., HPLC) are needed in order to identify possible active constituents responsible for photoprotective effects of the plant extracts.

The mechanisms by which AVS022 extracts suppressed activation of MMP-1 in HaCaT cells exposed to UVA were probably attributed to the attenuation of UVA-mediated ROS accumulation as a result of the augmentation of endogenous antioxidant capacity and did not involve the direct effects of the herbal extracts on the cells because treatment with the formula or each component herbs alone for 30 min in the absence of UVA irradiation did not substantially affect MMP-1 activity (data not shown). Additionally, inhibition of UV-induced MMP-1 activity and expression by natural products derived from medicinal plants could be regulated by multiple signal pathways including AP-1 and NF-kappa B transcription factors and MAP kinase [8, 14, 20]. Quercetin, a polyphenol commonly found in diet and medicinal plants, was demonstrated to block photocarcinogenesis in epidermal JB6 cells through downregulation of AP-1, NF-kappa B, and MAPK activities as well as activation of antioxidant transcription factor [21]. Further study is, thus, needed to explore an association between MMP-1 mediated by MAP kinase and redox modulation at molecular levels in keratinocytes exposed to UV irradiation. 

In conclusion, protective mechanisms by which AVS022, an oriental herbal formula, and its herbal components exerted inhibitory effects on UVA-induced MMP-1 activity involved regulation of endogenous antioxidants including GSH, catalase, and GPx. Additionally, antioxidant potential of the component herbs, particularly *F. racemosa*, and several phenolic compounds (e.g., GA) may be contributed to the pharmacological actions of AVS022 formula. This study could provide pharmacological evidence for polyherbal formula and its constituent herbs. Further identification of active compounds to validate biological activities of the formula is needed in order to develop the herbal formula containing antioxidant phytochemicals as effective photoprotective agents.
